# Charcot-Leyden Crystals in Hepatic Abscess: A Diagnostic Clue Unveiling Parasitic Infection Through Fine-Needle Aspiration Cytology

**DOI:** 10.7759/cureus.81947

**Published:** 2025-04-09

**Authors:** Anurag Singh, Ankita Singh, Pallavi Srivastava, Pallavi Prasad

**Affiliations:** 1 Pathology, Sanjay Gandhi Postgraduate Institute of Medical Sciences, Lucknow, IND

**Keywords:** charcot leyden crystals, entamoeba histolytica infection, fine-needle aspiration cytology, hepatic abscess, parasitic infection

## Abstract

Charcot-Leyden crystals (CLCs) are hexagonal, colorless, bipyramidal structures derived from the aggregation of materials formed by disintegrating eosinophils. Necrotic cell debris and CLCs serve as indirect indicators of parasitic infestation. Here, we highlight a case of a 49-year-old male patient who presented with high-grade fever and right upper quadrant abdominal pain. Radiological findings showed a hypodense liver lesion consistent with an abscess. Fine-needle aspiration cytology (FNAC) demonstrated necrotic debris and a significant presence of CLCs, with no identifiable parasites. Laboratory investigations omitted bacterial and fungal infections, while enzyme-linked immunoassay (ELISA) identified IgG antibodies for* Entamoeba histolytica*. A definitive diagnosis of amoebic liver abscess was rendered, and the patient was treated successfully with metronidazole and luminal amoebicides. This case report emphasizes the diagnostic importance of CLCs in hepatic aspirates, particularly when the direct demonstration of parasites is a challenge. CLCs may provide indirect evidence of parasitic liver abscesses despite negative results from stool and cytological examinations for parasites. Effective identification and evidence-based anti-amoebic treatment are essential in endemic areas to avoid superfluous examinations for neoplastic causes.

## Introduction

Charcot-Leyden crystals (CLCs), elongated bipyramidal hexagonal crystals, were first described by Jean-Martin Charcot in 1853, before Paul Ehrlich's discovery of eosinophils. At present, CLCs are acknowledged as a basic feature of eosinophilic degradation. The CLC protein has palmitate-cleaving lysophospholipase activity and is classified within the S-type lectin family, particularly galectin-10. CLC presence has consistently been linked with lytic eosinophils. A recent study reveals that cytolysis represents the occurrence of extracellular trap cell death (ETosis), an active non-apoptotic cell death process that liberates filamentous chromatin complexes [1]. Galectin-10 is a major protein found in the cytoplasm of eosinophils, although it is absent from secretory granules. Activated eosinophils undergo ETosis, resulting in the translocation of galectin-10 from the cytoplasm and the formation of intracellular CLC. Following the breakdown of the plasma membrane, free galectin-10 is released and forms extracellular CLCs [2]. The presence of CLCs, accompanied by an eosinophilic infiltrate, serves as an indirect indication of parasite infestation [3,4]. This rare case study demonstrates the presence of CLCs and eosinophilic infiltration in the hepatic aspirate.

## Case presentation

A 49-year-old male patient with known diabetes for the last year, controlled on oral hypoglycaemic drugs (Tab dapagliflozin and Tab metformin 500 mg BD), presented with intermittent high-grade fever (102.5°F) with chills and rigors for seven days. The patient also had pain in the right upper quadrant of the abdomen for the last four days. There was no history of nausea, vomiting, loose stool, or urinary symptoms. On examination, he was conscious, alert, and febrile (101.2°F). His pulse rate was 90/min, and his blood pressure was 120/78 mmHg. Mild pallor and icterus were noted. There was no clubbing, lymphadenopathy, or pedal oedema. The rest of the systemic examination was normal. On palpation, there was right upper abdominal tenderness; however, no clinical ascites was identified. His complete blood count analysis was within normal range, except for the total leukocyte count. The liver function test, renal function test, and coagulation study results were unremarkable. No elevation in tumor markers was seen (Table 1).

**Table 1:** Results of laboratory findings in a case of liver abscess at the time of presentation

Test name	Result	Reference range
Hematological findings		
Hemoglobin (g/dL)	15.0	12.5-15.5
Total leukocyte count (/L)	11.4 x 10^9^	4 x 10^9^-11 x 10^9^
Differential leukocyte count	Neutrophils, 85%	Neutrophils: 40-60%
	Lymphocytes, 11%	Lymphocytes: 20-40%
	Eosinophils, 3%,	Eosinophils: 1-4%
	Monocytes, 1%	Monocytes: 2-8%
Platelets (/L)	150 x 10^9^	150 x 10^9^-450 x 10^9^
Erythrocyte sedimentation rate (ESR)	25 mm at the first hour	<15 mm at the first hour
Coagulation profile findings		
Prothrombin time (sec)	14.5	12.5-13.7
Activated partial thromboplastin time (sec)	33.5	25.5-34.5
Fibrinogen (mg/dL)	390	180-450
Liver function test		
Total bilirubin (mg/dL)	0.95	0.2-1.2
Direct bilirubin (mg/dL)	0.37	0.1-0.8
Alkaline phosphatase (U/L)	105.2	25-110
Aspartate aminotransferase (IU/L)	36	12-40
Alanine aminotransferase (IU/L)	22	10-45
Renal function test		
Serum urea (mg/dL)	35	15-45
Creatinine (mg/dL)	0.95	0.5-1.5
Tumor markers		
Serum alpha-fetoprotein (ng/ml)	2.1	<10
Serum carcinoma embryonic antigen (ng/ml)	1.3	<3

**Figure 1:**
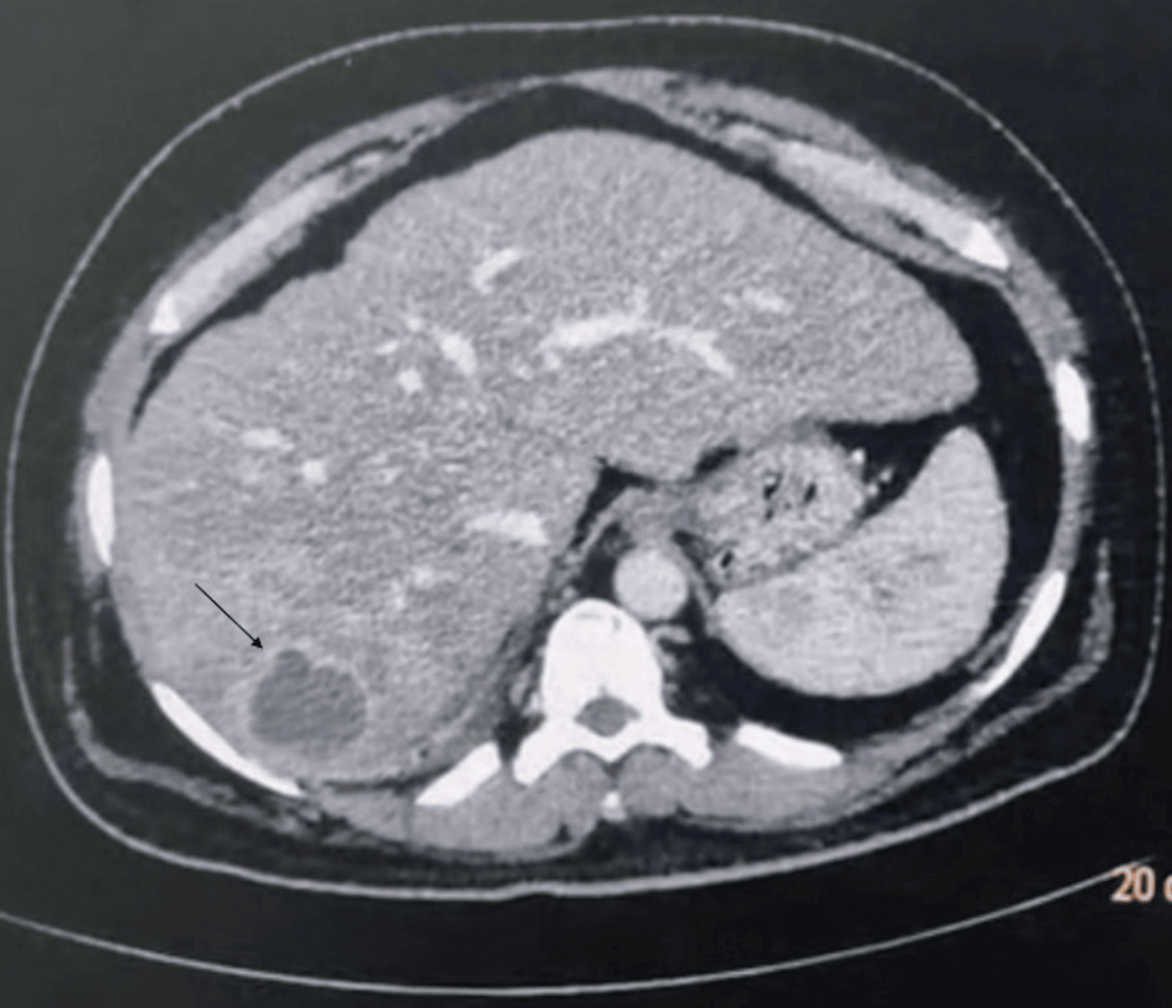
A contrast-enhanced computerized tomography scan of the abdomen showing a well-defined hypodense lesion in the right lobe (→) of the liver

Ultrasonographic (USG) examination of the whole abdomen revealed altered coarse echotexture of the liver, accompanied by uneven liver borders. An ill-defined hypoechoic lesion measuring 4.5 x 3.5 cm was noted with internal hyperechoic areas. USG findings are suggestive of liver abscess or primary hepatic neoplasm. A contrast-enhanced computed tomography (CECT) scan of the abdomen was performed to narrow down the diagnosis. The CECT scan showed the liver having irregular margins and a well-defined hypodense lesion in the right lobe, measuring 5.5 x 3.8 cm with minimal peripheral rim enhancement. The lesions showed no enhancement in the arterial phase and were better delineated in the venous and delayed phases. The possibility of liver abscess due to infectious etiology was kept. No evidence of intrahepatic biliary radical (IHBR) dilatation was seen. The gallbladder was well distended with normal wall thickness. There is no evidence of any radio-opaque calculi. No intraluminal mass or pericholecystic fluid was seen (Figure 1).

The patient subsequently underwent CT-guided aspiration of the liver lesion. On aspiration, 20 ml of blood-mixed pus-like material was aspirated. Liver pus aspiration was sent for cytological examination to look for malignant cells, parasites, granuloma, and acid-fast bacilli. A bacterial and fungal culture of the liver pus aspirate was also requested. The patient was managed with intravenous analgesics and antipyretics.

**Figure 2:**
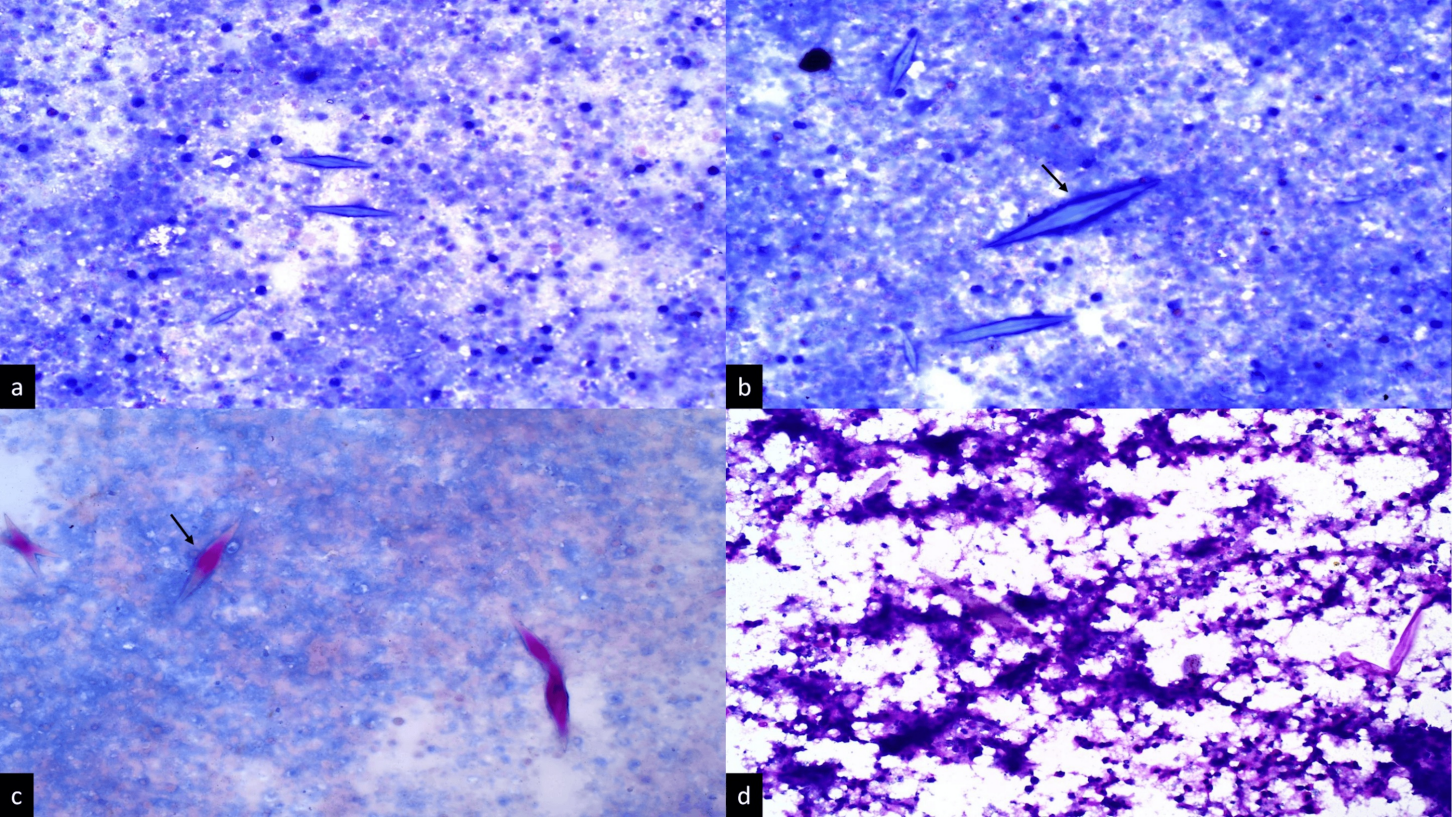
(a) A ultrasound-guided fine-needle aspiration smear from liver abscess displaying necrotic cell debris (Giemsa stain, x10). (b) High-power view photomicrograph showing Charcot-Leyden crystals (→) in the background (Giemsa stain, x40). (c) Ziehl-Neelsen stain showing highlighted Charcot-Leyden crystals (→) (x40). (d) No fungal elements are seen in Periodic acid-Schiff stain (x40)

USG-guided fine-needle aspiration cytology (FNAC) from the lesion was performed to make a definitive diagnosis. FNAC smears showed necrotic cell debris and numerous hexagonal, needle-shaped CLCs in the background; however, there were no parasites seen (Figures 2a, 2b). The Ziehl-Neelsen stain for acid-fast bacilli was negative, highlighting the hexagonal CLCs (Figure 2c). Periodic acid-Schiff (PAS) stain was also performed to rule out fungal infection, which showed negative results (Figure 2d).

Bacterial and fungal cultures of liver abscesses were sterile. No ova or cysts were found in the stool of the patient. Because of necrotic cell debris and CLCs, a possibility of parasitic infestation was suspected. Enzyme-linked immunosorbent assays (ELISAs) for IgG antibodies for *Entamoeba histolytica*, *Echinococcus granulosus, *and *Fasciola* species were performed. IgG antibodies for *Entamoeba histolytica* were found positive, and the rest were negative. The patient was treated with Tab Metrogyl 750 mg TID for 15 days, followed by a luminal amoebicidal agent (paromomycin, 30 mg/kg/day orally for seven days in three divided doses). After completion of treatment, his routine hemogram showed a notable improvement (Table 2). The patient became afebrile, pain was controlled, and he was discharged in stable condition.

**Table 2:** Results of hematological findings after completion of treatment

Test name	Result	Reference range
Hemoglobin (g/dL)	15.0	12.5-15.5
Total leukocyte count (/L)	6.2 x 10^9^	4 x 10^9^-11 x 10^9^
Differential leukocyte count	Neutrophils, 67%	Neutrophils: 40-60%
	Lymphocytes, 30%	Lymphocytes: 20-40%
	Eosinophils, 2%	Eosinophils: 1-4%
	Monocytes, 1%	Monocytes: 2-8%
Platelets (/L)	150 x 10^9^	150 x 10^9^-450 x 10^9^

## Discussion

The diagnosis of liver abscess is considered in individuals presenting with right upper abdominal pain associated with fever and generalized weakness. Infectious liver abscesses have a diverse etiology, the most common being amoebic, followed by pyogenic and helminthic liver abscesses. FNAC of these lesions shows necrotic cell debris, inflammatory cell infiltrate, and tissue necrosis. Less frequently, CLCs may be seen in the background of the smear [3,4].

FNAC smear displaying plenty of CLCs in a background of necrotic cell debris, predominantly of eosinophils, suggests a diagnosis of parasitic origin. Parasitic liver abscesses occur due to *Entamoeba histolytica*, visceral larva migrans, *Fasciola *species, *Ascaris lumbricoides*, *Toxocara *species, etc. Most of the cases remain subclinical but may be symptomatic as well. A CECT scan may show solitary or multiple hypoechoic lesions in the liver with or without hepatomegaly. A long-standing high eosinophil count is a diagnostic feature of helminthic infestation [4,5]. In the present case, the patient presented with fever and right upper quadrant abdominal pain and showed a solitary liver abscess on a CECT scan. There was no peripheral blood eosinophilia noted.

A hepatic lesion and peripheral leukocytosis with neutrophilia may be diagnostic problems when the stool examination and fine-needle aspiration smears revealed no parasites, and the bacterial and fungal culture was also sterile [4,6]. In adults presenting with similar clinical features of liver disease, a differential diagnosis may include *Entamoeba histolytica *infestation and helminthic infestation; however, inflammatory pseudotumor, Langerhans cell histiocytosis, and neoplastic lesions were not excluded based on radiology. Hence, to make a definitive diagnosis, FNAC from a liver lesion is significant. Based on the presence of necrotic cell debris along with CLCs and the absence of atypical cells, we can rule out the differential diagnosis of neoplastic lesions [6,7].

The diagnosis of Langerhans cell histiocytosis was ruled out since there were no characteristic histiocytes with folded or grooved nuclei, despite the presence of several CLCs with necrotic cell debris [8]. Finally, considering the radiological examination, fine-needle aspiration findings from the hepatic lesion, and the absence of eosinophilia in peripheral blood, a provisional diagnosis of *Entamoeba histolytica* was made. The ELISA test for* Entamoeba histolytica* IgG antibody was also found positive in the present case. In many cases of liver abscess, no parasite was demonstrated in the FNAC smears as well as by stool examination; still, a probable diagnosis of parasitic infestation should be kept in mind, particularly in the Asian population [9]. CLCs linked to eosinophils are commonly found in the stool samples of patients with amoebic enteritis. Nevertheless, they are infrequently found in the pus from an amoebic liver abscess, which primarily comprises necrotic inflammatory debris, eosinophils, and, on rare occasions, trophozoites of *Entamoeba histolytica *[10].

## Conclusions

The concept of ETosis is brought to light in this case study, which demonstrates the significant outcome of disintegrated cellular debris and numerous CLCs in fine-needle aspiration smears obtained from a hepatic lesion. It is possible to use the presence of CLCs as an indirect sign of parasitic infestation when there are no visible parasites present during the stool and cytological examination. In clinical practice, it is essential to evaluate an infectious aetiology, such as *Entamoeba histolytica*, in hepatic lesions before looking into a neoplastic differential. This is especially important in developing nations like India, where parasitic infestation is widespread. It is possible to greatly improve patient outcomes and reduce morbidity associated with liver abscesses caused by parasites by immediately diagnosing the condition and providing appropriate treatment.
